# The Application of Principal Component Analysis (PCA) for the Optimization of the Conditions of Fabrication of Electrospun Nanofibrous Membrane for Desalination and Ion Removal

**DOI:** 10.3390/membranes11120979

**Published:** 2021-12-13

**Authors:** Khaled Younes, Omar Mouhtady, Hamdi Chaouk, Emil Obeid, Rabih Roufayel, Ahmad Moghrabi, Nimer Murshid

**Affiliations:** College of Engineering and Technology, American University of the Middle East, Kuwait; omar.mouhtady@aum.edu.kw (O.M.); Hamdi-Chaouk@aum.edu.kw (H.C.); emil.obeid@aum.edu.kw (E.O.); rabih.roufayel@aum.edu.kw (R.R.); ahmad.moghrabi@aum.edu.kw (A.M.); nimer.murshid@aum.edu.kw (N.M.)

**Keywords:** desalination, principal component analysis, electrospun nanofibrous membrane (ENM), manufacturing conditions

## Abstract

Nowadays, acquiring a water supply for urban and industrial uses is one of the greatest challenges facing humanity for ensuring sustainability. Membrane technology has been considered cost-effective, encompasses lower energy requirements, and at the same time, offers acceptable performance. Electrospun nanofibrous membranes (ENMs) are considered a novel and promising strategy for the production of membranes that could be applied in several treatment processes, especially desalination and ion removal. In this study, we apply an unsupervised machine-learning strategy, the so-called principal component analysis (PCA), for the purpose of seeking discrepancies and similarities between different ENMs. The main purpose was to investigate the influence of membrane fabrication conditions, characteristics, and process conditions in order to seek the relevance of the application of different electrospun nanofibrous membranes (ENMs). Membranes were majorly classified into single polymers/layers, from one side, and dual multiple layer ENMs, from another side. For both classes, variables related to membrane fabrication conditions were not separated from membrane characterization variables. This reveals that membranes’ characteristics not only depend on the chemical composition, but also on the fabrication conditions. On the other hand, the process conditions of ENM fabrication showed an extensive effect on membranes’ performance.

## 1. Introduction

Membrane separation has attracted a lot of attention, being an efficient technique to suffice the urgent need to find low-cost and environmentally-friendly alternatives to the water treatment methods that were being studied. Membrane technology has already provided promising and reliable salt/contaminant/ion removal and freshwater production [1,2]. Several advantages arise from this technology, as it is considered more selective and requires less energy consumption, smaller systems, and a lower carbon footprint. These advantages have been highlighted by membranes and membrane processing, which have been intensively studied and improved over the past decades by industry professionals in water/wastewater treatment and desalination [3]. The driving force and membrane structure are the main factors that define how membrane processes are classified. Based on these factors, as well as the pore sizes, traditional membrane technologies for water treatment are mostly pressure-driven. These include microfiltration (MF), ultrafiltration, UF, nanofiltration (NF), and reverse osmosis (RO). All of these are isothermal processes [4,5]. RO and NF are primarily used for seawater desalination as well as ion removal from aqueous streams. The MF and UF processes are widely used for water treatment, particulate (mostly organic) removal from surface waters, and wastewater treatment. These membranes have been implemented as pretreatments for RO-based seawater desalination [1,6].

As previously mentioned, the applied membrane is a key component in all membrane processes. The fabrication method and the material used determine the intrinsic characteristics of the membrane. There are many methods to fabricate commercial membranes for water treatment or desalination. These include phase-inversion, sintering, and stretching, as well as track-etching. The most popular is the phase inversion technique using interfacial polymerization in the presence of polymer synthesis. This technique is particularly useful for RO and NF membranes [7]. Different membrane structures can be achieved using each technique. Thin-film membranes with nanocomposite structures are used mainly in commercial water desalination. They can be prepared using combined phase conversion and interfacial polymerization technologies [8]. Conventional interfacial polymerization remains the most popular method to make thin-film RO and NF membranes. New interfacial polymerization methods are being developed at the laboratory scale. They can be used to solve common problems, such as the trade-off between water permeability, solute rejection, controlled surface roughness, and large amounts of solvent usage. It is also difficult to embed nanomaterials in the selective layer [9].

It is important to note that membranes must be used with the highest level of selectivity and permeability due to the increasing complexity and quantity of contaminants in water resources. This has created a new demand for novel and innovative fabrication techniques. Electrospun nanofibrous membranes (ENMs) have been the focus of intensive research in water treatment and desalination [10,11]. Comparable to conventional fabrication techniques that were thoroughly tabulated in the literature [12], ENMs consist of randomly overlapping fibers ranging from several micrometers. A viscoelastic solution of a polymer solution is required to form one nanofiber in the electrospinning method. Uniaxial stretching occurs under a high-voltage field. These ENMs are distinguished by their unique characteristics, such as a high surface-to volume ratio, high porosity, and interconnected pores with 3-D structures. These properties are due to a unique architecture made from randomly collected nanofibers. ENMs are widely considered a viable alternative to conventional medical equipment in a variety of applications, such as healthcare, medical stuff, food packaging, and environmental issues. ENMs are considered a promising candidate for membrane technology [13].

**Figure 1:**
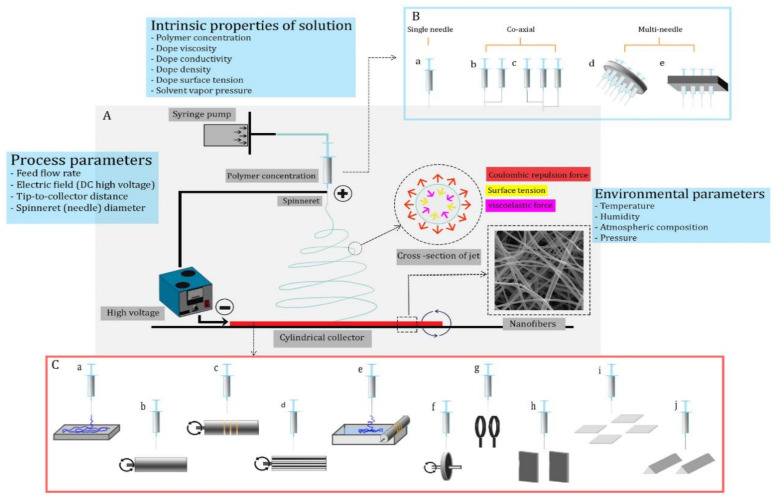
(**A**) Electrospinning process’ elementary principles, the main counterparts of a conventional setup, and influencing parameters and forces; Coulombic repulsion force, surface tension, and viscoelastic force represent the forces acting on the solution jet. (**B**) Different kinds of spinnerets involved in electrospinning for ENM fabrication. (**C**) Types of collectors for providing different nanofiber orientations and arrangements (a: flat plate, b: rotary drum, c: rotary drum with wrapped copper wire, d: rotary wire drum, e: water bath, f: rotating disk, g: parallel rings, h: static blade electrodes, i: counter electrode collector, and j: long parallel electrodes) (from Sanaeepour et al. [22]).

In the 1990s, electrospinning technology was introduced. The process and its applications have seen significant improvements in the past few years. Figure 1 shows a typical schematic of an electrospinning system. It can be divided into four parts: an injection system for polymer solution, a spinneret supply, a grounded conductive collector, and a high-voltage supply. A liquid jet (polymer-dope solution) is required to lengthen a single fiber within the range of a few micrometers. Three general stages of nanofiber formation can be distinguished: (1) the appearance of jetting and its development (i.e., formation of Taylor’s cone); (2) bending with wavy motion (spiraling path and looping path); and (3) random collection of nanofibers [4,14]. The formation of Taylor’s cone is strongly affected both by the solution injection rate and the DC voltage between spinneret/collector. A DC electrical potential difference must be created between the spinneret tip (where Taylor’s cone was formed from the dope solution) and the collector (Figure 1B). The critical voltage for nanofiber generation strongly influences the solution’s surface tension, spinneret geometry, and spinning distance (a distance from the spinneret tip to the collector) [15,16]. The solution jet travels straight up the collector surface after initiating electrospinning. It then spirals and loops towards the collector surface. The applied voltage and solution conductivity are all factors that affect the jet’s movement in this area. It is, however, inversely proportional to the current passing through the jet and the density of the polymer solution. This step results in the solidification and evaporation of the solvent. The collector can then be used to form nanofibers and deposit them randomly. The final morphology and geometry of nanofibers will depend on the design of the collector [12,17,18,19,20,21]. 

The above discussion shows that electrospinning presents an excellent and promising technology for fabricating highly efficient membranes. It can be used as an individual membrane or as a selective layer with unique features for desalination and ion removal from a water medium. After overseeing the principles of electrospinning, it can be noticed that different operating conditions can have an impact on the morphology and appearance of the nanofibers. These parameters can be divided into three groups: membrane fabrication, characterization, and performance [10]. These factors will be the heart of our discussion in this study. Tables S1 and S2 present a set of published results on single and multiple layer/polymer ENMs for water treatment (desalination and ion removal), respectively (summarized by Sanaeepour et al. [22]). Understanding the interaction between operating parameters and membrane characteristics and features is considered tedious and time consuming [23]. In order to simplify this, a sophisticated data mining technique can be adopted, such as principal component analysis (PCA). In this study, PCA has been applied to Tables S1 and S2, separately, to investigate the influence of membrane fabrication conditions, characteristics, and process conditions, and in order to seek the relevance of the application of different electrospun nanofibrous membranes (ENMs).

## 2. Principal Component Analysis

PCA represents a method of analyzing relationships among variables within a system if all the important variables are known. It is classified as an unsupervised machine-learning method in the sense that no previous knowledge or output is anticipated by the user [24]. It groups the operating variables to determine their main influencing variables [24,25,26,27]. PCA can be used to identify the main variables and their effect on the target population of the dataset. Karl Pearson introduced PCA in 1901 [28,29]. It is also known as the discrete Karhunen Loeve transform (KLT). The PCA technique is used to reduce the problem’s dimensionality by making a large number of input variables smaller. These are called principal components (PCs). The accuracy of the problem can be affected by reducing the number of variables. There is, therefore, a compromise between simplicity and accuracy. The PCA approach has a primary goal to simplify the problem and preserve as much accuracy as possible. It reduces the number of variables, but retains most of the information from the input features. PCA is a statistical process that transforms the potentially correlated input features into uncorrelated variables. PCA reduces the problem’s dimensionality by projecting the observation data orthogonally onto the lower-dimensional subspace PCs. This allows for a lower-dimensional problem and preserves accuracy.

In the desalination process, PCA was used to investigate the effect of transmembrane pressure (TMP) on fouling during the ultrafiltration process (UF) [24,30], as the effects of water characteristics on the efficiency of filtration were studied in reverse osmosis (RO). A process prediction model was then developed using an artificial network [30]. The following provides a detailed description of the PCA methodology adopted in this study. The ith PC matrix (*Fi*) is expressed using a unit-weighting vector (*Z_i_*) and the original data matrix *Y* with *p* × *n* dimensions. (*p*: number variables, *n*: number of datasets) as follows [24]:

(1) $$Fi=Z_{i}^{T}Y=\sum_{}^{j=0}z_{ij}y_{j}$$

where *z* is the loading coefficient and *y* is the data vector of size *n*. The variance matrix *Y(Var(Y)*), is obtained by projecting *Y* to *Z* and should be maximized, as follows:

(2) $$Var\left(Y\right)=\frac{1}{n}\;\left(ZY\right){\left(ZY\right)}^{T}=\frac{1}{n}\;ZYY^{T}Z$$

(3) $$MaxVar\left(Y\right)=Max\left(\left(\frac{1}{n}\right)\;ZYY^{T}Z\right)$$

Since $\frac{1}{n}\;YY^{T}$ is the same as the covariance matrix of *Y(cov(Y))*, *Var(Y)* can be expressed, as follows:

(4) $$Var\;\left(Y\right)=Z^{T}cov\;\left(Y\right)\;Z\;$$

The Lagrangian function can be defined by performing the Lagrange multiplier method, as follows:

(5) $$L=Z^{T}cov\left(Y\right)Z-\delta \left(Z^{T}Z-1\right)$$

The term “*ZTZ*^−1^” is considered to be 0 in Equation (5), since the weighting vector is a unit vector. Hence, the maximum value of *var(Y)* can be calculated by equating the derivative of the Lagrangian function (*L*) with respect to *Z*, as follows:

(6) $$\frac{dL}{dZ}=0$$

(7) $$cov\left(Y\right)Z-\delta Z=\left(cov\left(Y\right)-\delta I\right)Z=0$$

where “*Z*” is the eigenvector of “*cov(Y)*” and δ is the eigenvalue of “*cov(Y)*”. The percent of the variance explained can be yielded using the eigenvalue ratio of the ith PC in respect to the overall dataset; this proportion indicates the extent to which the PC(s) cover of the total variance of the investigated dataset. Reduction in the dimensions of the variables is achieved through PCA by identifying the number of PCs less than *p*, which represents the dataset through the explained variance.

The data of each of the investigated variables has a different weight. In order to calibrate the variables’ influence on the dataset, a standardization technique is required, and “*Yst*“ is identified as follows:

$$Y_{st}=\frac{\left(Value-Mean\right)}{Standard\;Deviation}$$

After data standardization, PCA results were obtained using XLSTAT 2014 software, following the strategy adopted by Younes et al. [31,32,33,34]. Some data in Tables S1 and S2 were not discussed. In these cases, the missing data were estimated using a built-in function that replaces the missing value with the mean of each factor investigated.

## 3. Results and Discussion

**Figure 2:**
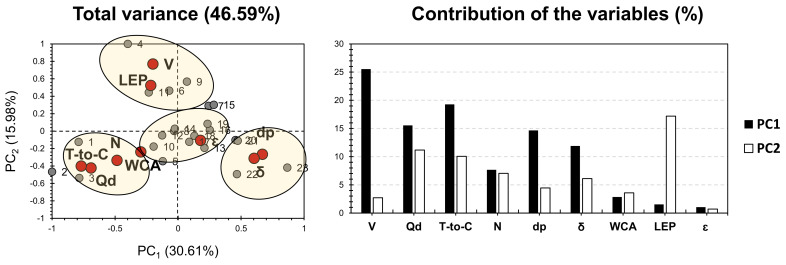
PCA for the results of single layer/polymer ENMs (by Sanaeepur et al. [22]; see Table S1). Grey bullets represent the 23 investigated membranes; Red bullets represent the involved variables (V: high voltage (kV), LEP: liquid entry pressure (kPa), WCA: water contact angle (o), ε: porosity (%), dp: pore diameter (μm), δ: thickness (μm), N: needle (spinneret; mm), T-to-C: tip-to-collector distance (cm), Qd: dope injection rate (mL·h^−1^)).

Figure 2 shows the PCA bi-plot for the published results on single polymer/layer ENMs for desalination and ion removal, shown in Table S1. Hence, the variables taken into account can be sub-divided into two main parts: membrane fabrication conditions and membrane characteristics and properties (Table S1). PCA accounted for 46.59% of the total variance. For the factors, V, Qd, T-to-C, dp, and δ exhibited the highest contribution along PC1, accounting for 87% of its weight. As for PC2, LEP exhibited the highest contribution, accounting for 17% of its weight. Pore size (dp; µm) and thickness (δ; µm) exhibited a positive influence on PC1 and a slightly negative influence, following PC2. The tip-to-collector distance (T-to-C), dope injection rate (Qd), needle (spinneret) diameter (N), and water contact angle (WCA) showed a negative influence on both PCs. Liquid entry pressure (LEP) and high voltage (V) presented a high positive and a slight negative influence, following PC2 and PC1, respectively. Interestingly, the variables related to membrane fabrication were not separated from membrane characterization variables. This reveals a high indication that the membrane characteristic does not only depend on the membrane chemical composition, but also on the fabrication conditions. The latter therefore requires attention, for the sake of enhancing membrane efficiency.

For the variables, four different clusters can be distinguished with respect to the disparity that variables hold, following their fabrication methods and characteristics. Variables of cluster 1 exhibited a high positive correlation with dp and δ. This indicates that the major factors influencing membranes composing cluster 1 are the size and textural conditions. In other words, the physical and textural properties of these membranes should be taken into consideration carefully for the sake of seeking their highest efficiency. Interestingly, the common features that can be noticed between the membranes of cluster 1 are the PVDF-based P-layer and acetone added to the S-layer. For cluster 2, its variables exhibited a high positive correlation with V and LEP. It is noteworthy that membranes of this cluster do not share any doping feature, either from the S- or P-layers. This probably highlights some influence of the voltage implemented in the membrane’s fabrication on the membrane’s permeability. The latter is counted as the main factor influencing LEP. For cluster 3, its variables showed a high correlation with T-to-C, N, WCA, and Q. Nonetheless, no explanation can be given in regard to the proximity noticed for membranes composing cluster 3 (membranes 1, 2, and 3). The only common feature is DMF being a part of or exclusively forming the S-layer. Nonetheless, this feature is shared with other membranes that are not part of cluster 3. Cluster 4 compiles the largest number of membranes, and it is well correlated with the porosity ε. It is worth mentioning that cluster 4 membranes are more likely positioned around the node between PC1 and PC2. This indicates a minor influence of these membranes on the multi-dimensional analysis adopted. For that purpose, it is valid to exclude membranes of cluster 4 in order to seek better intercorrelation between different factors, and therefore enhance discrepancy and similarity sight of the investigated membranes (other than cluster 4 membranes).

**Figure 3:**
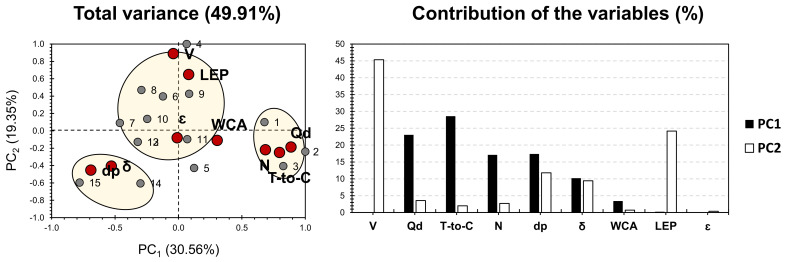
PCA for the results of single layer/polymer ENMs, after discarding membranes that are mostly influenced by porosity (by Sanaeepur et al. [22]; see Table S1). Grey bullets represent the 23 investigated membranes; Red bullets represent the involved variables (V: high voltage (kV), LEP: liquid entry pressure (kPa), WCA: water contact angle (o), ε: porosity (%), dp: pore diameter (μm), δ: thickness (μm), N: needle (spinneret; mm), T-to-C: tip-to-collector distance (cm), Qd: dope injection rate (mL·h^−1^)).

Figure 3 shows the PCA bi-plot for the dataset shown in Table S1, after discarding membranes that are mostly influenced by porosity (most of the membranes of cluster 4, in Figure 2). PCA accounted for a slightly higher variance, showing 49.91% (Figure 3) rather than 46.58% (Figure 2). This ensures a 3% higher reliability of the dataset treated in PCA of Figure 2. For the factors Qd, T-to-C, dp, and N exhibited the highest contribution along PC1, accounting for nearly 80% of its weight. As for PC2, LEP, and V exhibited the highest contribution, accounting for 70% of its weight. Even though factors revealed similar intercorrelations as the ones yielded in Figure 2, different behavior was observed along the first two PCs. Accordingly, LEP and V exhibited no influence on PC1, along its major positive influence, along PC2, and as highlighted in the first findings. For the other factors, their PCs’ influence was nearly inverted, as dp and δ showed a negative influence along both PCs. Qd, N, and T-to-C exhibited a positive influence on PC1, with a negative influence on PC2. Interestingly, WCA was discarded from the latter factor group and showed proximity to ε. For the variables, and similar to the first PCA findings (Figure 2), four different clusters have been implemented. Even though both PCAs showed the same clustering capacity, the distribution along clusters has been drastically modified. Both PCAs of the dataset, shown in Table S1, exhibited approximately the same variance along the first two PCs. The major contributors were Qd and T-to-C. Therefore, for the investigated membranes, special care should be taken for these two factors when developing a membrane and choosing the proper doping strategy. Once the membranes of cluster 4 (Figure 3) were discarded, it can be noticed that membranes were even more influenced by the electrospinning process parameters. 

**Figure 4:**
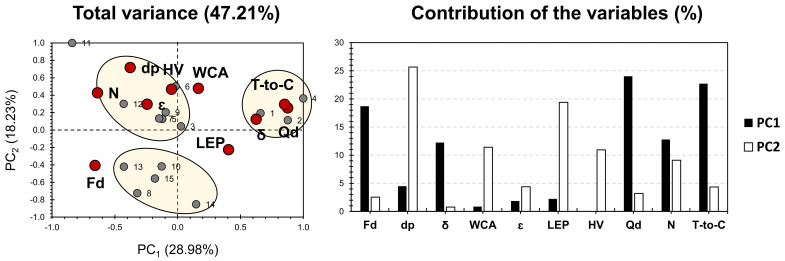
PCA for the results of dual and triple layer/polymer ENMs (by Sanaeepur et al. [22]; see Table S2). Grey bullets represent the 23 investigated membranes; Red bullets represent the involved variables (V: high voltage (kV), LEP: liquid entry pressure (kPa), WCA: water contact angle (o), ε: porosity (%), dp: pore diameter (μm), δ: thickness (μm), N: needle (spinneret; mm), T-to-C: tip-to-collector distance (cm), Qd: dope injection rate (mL·h^−1^), Fd: fiber diameter (nm)).

Figure 4 shows the PCA bi-plot for the published results on dual- and triple-layer ENMs for desalination and ion removal, shown in Table S2. The variables taken into account can be sub-divided into three main parts: membrane fabrication conditions, membrane characteristics, and performance. PCA accounted for 47.21% of the total variance. For the factors, Qd, T-to-C, and Fd exhibited the highest contribution along PC1, accounting for nearly 65% of its weight. As for PC2, LEP, dp, WCA, and V exhibited the highest contribution, accounting for 67% of its weight. WCA, dp, ε, HV, and N showed a high positive influence along PC2 and a relatively negative influence along PC1. This indicates that a membrane’s fabrication condition, V and N, are most likely influenced by the correlated membrane characteristics previously mentioned. LEP, δ, T-to-C, and Qd exhibited a high positive influence along PC1, with a minor fluctuating positive/negative influence, along PC2. Interestingly, Fd showed a low correlation with other factors and was negatively influenced by both PCs.

For the variables, three different clusters can be distinguished, in respect to the discrepancy that membranes hold, following their fabrication methods, characteristics, and performance. Variables of cluster 1 exhibited a high positive correlation with Dp, V, WCA, N, and ε. Interestingly, all of the membranes of this cluster (3, 5, 6, 7, 9, 12) have PVDF moieties in their composed layers, either on the top, middle, and/or the support. The variables of cluster 2 showed a high positive correlation with LEP, δ, T-to-C, and Qd. Interestingly, membranes 2 and 4 share the fact that HIPS moieties are present in the top layer (22–25 wt%; Table), and DMF was exclusively found in the membranes of cluster 2 (membranes 1, 2, and 4; Table). This probably reflects a certain relevance of employing HIPS and DMF on the positively correlated factors of cluster 2. Variables of cluster 3 showed a relatively lower positive correlation with Fd, compared to the correlation of populations of clusters 1 and 2, in respect to their allocated variables. This can be noticed by a higher distance between Fd and cluster 3 membranes than the distance noticed between cluster 1 and 3 variables, from one side, and their respective factors, from another side. Similarly, to single-layered membranes, the dual and multiple membranes exhibited more influence on the process parameters, rather than the influence of the intrinsic properties of the solution.

## 4. Conclusions

In this study, we have attempted to investigate the influence of membrane fabrication conditions, characteristics, and process conditions in order to seek the relevance of the application of different electrospun nanofibrous membranes (ENMs). Membranes were majorly classified into single polymers/layers, from one side, and dual multiple layer ENMs, from another side. For both classes, variables related to membrane fabrication conditions were not separated from membrane characterization variables. This reveals that membranes’ characteristics not only depend on the chemical composition, but also on the fabrication conditions.

For single-layered ENMs, PCA findings have shown a relationship between P-layer-based PVDFY and acetone added to the S-layer on the textural properties of membranes. Other trends showed a high correlation of some membranes, which are totally independent, on the chemical composition scale. This probably showed the high influence of the fabrication conditions on the membrane’s physical properties (case of the correlation between V and LEP). Quite a few membranes exhibited a near position around the node between PC1 and PC2. Once discarded, PCA findings (Figure 3) showed almost nearly inverted results, with a slightly higher variance (3%). This indicates that, even though PCA is an unsupervised machine learning tool, one should be attentive regarding the quality of data to be employed. Additionally, PCA findings and data trends strongly depend on the quality of the input data.

For the dual- and triple-layered ENMs, PCA findings have shown that PVDF moieties probably present an effect on Dp, WCA, and ε, from the physical properties side, in addition to N and V, from the membrane’s fabrication side. It also showed a certain relevance of employing HIPS and DMF on LEP, and δ, from the physical properties side, in addition to T-to-C and Qd, from the membrane fabrication strategy side.

In brief, PCA of single-layered ENMs showed high similarities between membranes that present membranes that presented lesser similarities at the chemical composition. This indicates that membrane fabrication conditions have an extensive effect on membrane performance. Although dual- and multiple-layered ENMs showed similar trends to single-layered ENMs, more effects of the matrix chemical composition on their physical properties have been noticed. For both types of membranes, the ENM fabrication showed an extensive effect on performance.

## Data Availability

Not applicable.

## Supplementary Materials

The following are available online at https://www.mdpi.com/article/10.3390/membranes11120979/s1, Table S1: Published results on single polymer/layer ENMs for desalination and ion removal (from Sanaeepur et al. [22]), Table S2: Published results on dual and triple layer ENMs for desalination and ion removal (membrane characterization; from Sanaeepur et al. [22]), Table S3: Published results on dual and triple layer ENMs for desalination and ion removal (membrane fabrication; from Sanaeepur et al. [22]), Table S4: Published results on dual and triple layer ENMs for desalination and ion removal (membrane performance; from Sanaeepur et al. [22])
