# Supplementary material for: The Application of Principal Component Analysis (PCA) for the Optimization of the Conditions of Fabrication of Electrospun Nanofibrous Membrane for Desalination and Ion Removal
Source: Membranes (Basel). 2021 Dec 13;11(12):979. doi: 10.3390/membranes11120979 (PMC8709082; doi:10.3390/membranes11120979)
Supplement: Supplementary file 1 [file membranes-11-00979-s001.zip › membranes-1497566-supplementary (1).pdf]

membranes

Supplementary Information

The Application of Principal Component Analysis (PCA) for the Optimization of the Conditions of Fabrication of Electrospun Nanofibrous Membrane for Desalination and Ion Removal

Khaled Younes *, Omar Mouhtady, Hamdi Chaouk, Emil Obeid, Rabih Roufayel, Ahmad Moghrabi and Nimer Murshid

College of Engineering and Technology, American University of the Middle East, Kuwait; omar.mouhtady@aum.edu.kw (O.M.); Hamdi-Chaouk@aum.edu.kw (H.C.); emil.obeid@aum.edu.kw (E.O.); rabih.roufayel@aum.edu.kw (R.R.); ahmad.moghrabi@aum.edu.kw (A.M.); nimer.murshid@aum.edu.kw (N.M.)

* Correspondence: Khaled.younes@aum.edu.kw

Table S1: Published results on single polymer/layer ENMs for desalination and ion removal (from Sanaeepur et al. [22])

Dope		V (kV)	Q_d (mL·h⁻¹)	T-to-C(cm)	N (mm)	d_p (μm)	δ (μm)	WCA (°)	LEP (kPa)	ε (%)
• P: PMMA (20 wt%) • S: DMF + acetone	1	18	9.6	30	-	0.41	161	164	227	83
• P: SAN (22.5 wt%) • S: DMF	2	18	16.2	30	0.68	0.18	105	133	118	70
• P: PS (18 wt%) • S: DMF	3	18	7.02	30	0.83	0.56	65	154	38	74
• P: PVDF + TBAC + FA • S: DMF + acetone	4	30	1	15	-	0.235	60	137	230	70
• P: PAN (10 wt%) • S: DMF	5	10	0.5	-	0.8	-	-	-	-	-
• M: keratin (15 wt%) • S: formic acid	6	25	0.06	20	0.2	-	50	-	-	90
• P: PAN (2,6, 10 &12 wt%) • S: DMF	7	17.5	0.6	10	0.2	-	-	-	-	-
• P: PTFE emulsion + PVA • S: Water	8	15	-	15	-	-	156	150	165	79.8
• P: matrimid (18 wt%) • S: NMP	9	30	1	17	0.26	2.25	-	130	-	-
• P: matrimid (25 wt%) • S: DMF + acetone	10	24	1.23	27.7	0.6	-	-	-	35	-
• P: PVDF (5-15 wt%_ • S: DMF +acetone	11	24	-	15	-	0.545	71	136	-	54
• P: PS (20 wt%) • S: DMF	12	18	0.1	17	0.83	0.235	-	145.5	-	75
• P: PS (18 wt%) • S: DMF	13	18	0.1	17	0.83	5.1	-	128	121	75
• P: PVDF (18 wt%) • S: DMF	14	18	2	18	-	0.32	150	-	-	76

**• P: PAN (10-12 wt%)**<br>**• S: DMF**	15	20	0.2	-	0.521	-	-	37	-	-
**• P: PAN (12 wt%)**<br>**• S: DMAc**	16	15	1	15	0.3	-	90	-	-	89
**• P: PAN (8-12 wt%)**<br>**• S: DMF**	17	16	0.9	-	0.6	0.55	190	130	-	-
**• P: PVDF (18 wt%)**<br>**• S: DMF**	18	18	2	18	-	-	150	-	-	-
**• P: PAN (10 and 15 wt%)**<br>**• S: DMF**	19	20	1	15	0.5	-	-	-	-	-
**• P: PVDF-HFP (14 wt%)**<br>**• S: DMF + acetone**	20	20	1	15	0.51	-	350	-	-	-
**• P: PVDF-HFP (14 wt%) + Polypyrrole**<br>**• S: DMF + acetone**	21	20	1	15	0.51	-	350	-	-	-
**• P: PSf (20 wt %)**<br>**• S: DMF**	22	12	4	15	0.21	2.9	135	-	12.4	-
**• P: PVDF (15 wt%)**<br>**• S: DMAc + acetone**	23	15	2	15	0.21	7.3	300	145	-	-

Table S2. Published results on dual and triple layer ENMs for desalination and ion removal (membrane characterization; from Sanaeepur et al. [22])

Membrane characterization							
**Layers**		**Fd (nm)**	**dp (μm)**	**δ (μm)**	**WCA (°)**	**ε (%)**	**LEP (kPa)**
**Top: SAN (17.5 wt%) + DMSO/acetone<br>Middle: -<br>Support: PAN (10 wt%) + DMF**	1	300	0.28	97	155	76	169
**Top: HIPS (22&25 wt %) + DMF<br>Middle: SBR (8 wt%) + DMF/THF<br>Support: PP**	2	601	0.43	160	154	71	181
**Top: FAS functionnalized PVDF-HFP<br>Middle: -<br>Support: PVDF-HFP**	3	-	0.49	111	160	86	187
**Top: HIPS (22 wt %) + DMF/acetone<br>Middle: -<br>Support: SAN (24 wt%) + DMF/acetone**	4	536.5	0.43	173	143	77.8	135.6
**Top: PVDF+PDMS<br>Middle: -<br>Support: PVDF-HFB**	5	-	0.43	88	170	77	129
**Top: PVDF+DMF/acetone<br>Middle: -<br>Support: nonwoven fabrics**	6	133	1.65	190	140	66	43.5
**Top: PDMS<br>Middle: -<br>Support: PVDF-HFP (15 wt%)**	7	-	0.49	102	155.4	87	126
**Top: PVA (4-8 wt%)<br>Middle: -<br>Support: PP**	8	-	0.08	48	105	-	-
**Top: PVDF-HFP (20 wt%)<br>Middle" -<br>Support: PVA, Nylon 6, PAN**	9	239.3	0.34	128	148	89	122.3
**Top: Chitosan + PVA (8 wt%)<br>Middle: -<br>Support: PAN**	10	932	0.6	-	-	87	-
**Top: PVDF-HFP (15 wt%)<br>Middle: -<br>Support: PAN (8 wt%)**	11	225	1.55	81	150	90	85.5

**Top: Silica + FS10**<br>**Middle: -**<br>**Support: PVDF**	12	180	0.83	72	150	80	150
**Top: Polyaniline**<br>**Middle: -**<br>**Support: PAN**	13	200	-	50	-	70	-
**Top: PVDF**<br>**Middle: PVDF**<br>**Support: PAN**	14	-	0.1	175	145	-	350
**Top: PVDF-HFP(10 wt%)**<br>**Middle: -**<br>**Support: PVDF-HFP**	15	200	0.26	110	126	60	131.6

Table S3. Published results on dual and triple layer ENMs for desalination and ion removal (membrane fabrication; from Sanaeepur et al. [22])

**Membrane characterization**		**Membrane fabrication (electrospinning)**				
**Layers**		**HV**	**Qd**	**N**	**T-to-C (cm)**	**Qa (NL.min-1)**
**Top: SAN (17.5 wt%) + DMSO/acetone**<br>**Middle: -**<br>**Support: PAN (10 wt%) + DMF**	1	18	5.4	18	30	2
**Top: HIPS (22&25 wt %) + DMF**<br>**Middle: SBR (8 wt%) + DMF/THF**<br>**Support: PP**	2	18	5.4	18	30	2
**Top: FAS functionnalized PVDF-HFP**<br>**Middle: -**<br>**Support: PVDF-HFP**	3	16	0.8	-	15	0-4
**Top: HIPS (22 wt %) + DMF/acetone**<br>**Middle: -**<br>**Support: SAN (24 wt%) + DMF/acetone**	4	20	8.1	18	35	3
**Top: PVDF+PDMS**<br>**Middle: -**<br>**Support: PVDF-HFB**	5	18	1.5	-	8	-
**Top: PVDF+DMF/acetone**<br>**Middle: -**<br>**Support: nonwoven fabrics**	6	16	1.5	18	15	-
**Top: PDMS**<br>**Middle: -**<br>**Support: PVDF-HFP (15 wt%)**	7	18	0.7	-	15	-

**Top: PVA (4-8 wt%)**<br>**Middle: -**<br>**Support: PP**	8	16	0.3	-	13	-
**Top: PVDF-HFP (20 wt%)**<br>**Middle" -**<br>**Support: PVA, Nylon 6, PAN**	9	21	0.8	-	20	-
**Top: Chitosan + PVA (8 wt%)**<br>**Middle: -**<br>**Support: PAN**	10	13	0.2	-	10	-
**Top: PVDF-HFP (15 wt%)**<br>**Middle: -**<br>**Support: PAN (8 wt%)**	11	20	1	21	15	-
**Top: Silica + FS10**<br>**Middle: -**<br>**Support: PVDF**	12	26	1.2	-	12	-
**Top: Polyaniline**<br>**Middle: -**<br>**Support: PAN**	13	14	1.2	-	10	-
**Top: PVDF**<br>**Middle: PVDF**<br>**Support: PAN**	14	16	2	-	15	-
**Top: PVDF-HFP(10 wt%)**<br>**Middle: -**<br>**Support: PVDF-HFP**	15	20	1	18	15	-

Table S4. Published results on dual and triple layer ENMs for desalination and ion removal (membrane performance; from Sanaeepur et al. [22])

**Membrane characterization**	**Membrane Performance**				
**Layers**	**Feed**	**delta T**	**Qf**	**Qp**	**F**
**Top: SAN (17.5 wt%) + DMSO/acetone**<br>**Middle: -**<br>**Support: PAN (10 wt%) + DMF**	35	40	0.48	0.24	37-84
**Top: HIPS (22&25 wt %) + DMF**<br>**Middle: SBR (8 wt%) + DMF/THF**<br>**Support: PP**	100	40	0.48	0.24	18.33
**Top: FAS functionnalized PVDF-HFP**<br>**Middle: -**<br>**Support: PVDF-HFP**	1 M NaCl + 20 mM CaCl2	40	30 L/h	30 L/h	0.9-1
**Top: HIPS (22 wt %) + DMF/acetone**<br>**Middle: -**<br>**Support: SAN (24 wt%) + DMF/acetone**	-	40	0.48	0.4	23.56

**Top: PVDF+PDMS**<br>**Middle: -**<br>**Support: PVDF-HFB**	-	40	-	-	36
**Top: PVDF+DMF/acetone**<br>**Middle: -**<br>**Support: nonwoven fabrics**	3.5 % NaCl solution	60	4800	4800	49.3
**Top: PDMS**<br>**Middle: -**<br>**Support: PVDF-HFP (15 wt%)**	-	40	0.5	0.5	34
**Top: PVA (4-8 wt%)**<br>**Middle: -**<br>**Support: PP**	-	20	-	-	7
**Top: PVDF-HFP (20 wt%)**<br>**Middle" -**<br>**Support: PVA, Nylon 6, PAN**	-	40	-	-	44484
**Top: Chitosan + PVA (8 wt%)**<br>**Middle: -**<br>**Support: PAN**	-	-	-	-	320
**Top: PVDF-HFP (15 wt%)**<br>**Middle: -**<br>**Support: PAN (8 wt%)**	35	40	-	-	30
**Top: Silica + FS10**<br>**Middle: -**<br>**Support: PVDF**	3.5 wt%	40	-	-	24.6
**Top: Polyaniline**<br>**Middle: -**<br>**Support: PAN**	-	-	-	-	-
**Top: PVDF**<br>**Middle: PVDF**<br>**Support: PAN**	3.5 wt%	60	-	-	16
**Top: PVDF-HFP(10 wt%)**<br>**Middle: -**<br>**Support: PVDF-HFP**	-	30	-	-	20-22
